# A C**omprehensive** Analysis of the Radiographic Characteristics and Bilateral Symmetry of the Mental Foramen

**DOI:** 10.1002/cre2.70081

**Published:** 2025-02-06

**Authors:** Radhwan Algabri, Faisal Abulohoom, Abdelrahman Fadag, Hesham Al‐Sharani, Sami Al‐Aqab, Nassr Al‐Hutbany, Emad Alryashi, Ahmed Keshk, Ahmed Yaseen Alqutaibi

**Affiliations:** ^1^ Department of Prosthodontics, Faculty of Dentistry Ibb University Ibb Yemen; ^2^ Department of Prosthodontics, Faculty of Dentistry National University Ibb Yemen; ^3^ Department of Oral and Maxillofacial Surgery, Faculty of Dentistry Ibb University Ibb Yemen; ^4^ Department of Oral and Maxillofacial Surgery, Faculty of Dentistry Jiblah University for Medical and Health Sciences Ibb Yemen; ^5^ Department of Endodontics, Faculty of Dentistry Ibb University Ibb Yemen; ^6^ National Center for Epidemiology and Population Health, ANU College of Health and Medicine Australian Capital Territory Australia; ^7^ Department of Prosthodontics, Faculty of Dentistry Mansoura University Mansoura Egypt; ^8^ Department of Prosthodontics and Implant Dentistry, College of Dentistry Taibah University Al Madinah Saudi Arabia

**Keywords:** anatomic position, genders, mental foramen, panoramic radiograph, sides, symmetry

## Abstract

**Objectives:**

There is currently a scarcity of data on the frequency and bilateral symmetry of the position and other characteristics of the mental foramen (MF) and accessory mental foramina in Yemen. The objective of this study was to analyze the position and other characteristics, as well as the symmetry of the MF, in a sample of the Yemeni population.

**Materials and Methods:**

A retrospective analysis was conducted on a sample of 500 digital panoramic radiographs (1000 sides). The study examined various characteristics of the MF, including horizontal and vertical positions, shapes, appearances, and the presence of accessory mental foramina. Additionally, the study explored potential associations between these characteristics and variables such as the subject's gender, sides, and symmetry. Data analysis was performed using SPSS, and statistical significance was evaluated using chi‐square tests; the *p* value was set at 0.05.

**Results:**

The horizontal position of the MF was most frequently observed between the first and second lower premolars (63.2%). The vertical position of the MF was predominantly below the apices of the lower premolars (66.2%). The majority of MFs had a round shape (46.3%). In 72% and 75.6% of cases, there was a continuous and descending relationship between the MF and the mandibular canal, respectively. Accessory mental foramina was present in 3.8% of cases. Gender differences were significant for the shape of the MF and the pattern of the canal on the right side. The symmetry rates for various features included horizontal positions (87.4%), vertical positions (82.6%), and shapes (80.4%).

**Conclusion:**

The MF is most commonly situated horizontally between the lower premolars and vertically below the apices of these teeth. The majority of MFs showed a round shape and a continuous and descending relationship with the mandibular canal. In most instances, characteristics of the MF were symmetrical on both sides.

## 1 Introduction

The surgical management of patients aims to restore both form and function while preserving critical anatomical landmarks necessary for planning surgical approaches. One such landmark that has gained significant attention is the mental foramen (MF), due to its importance in various dental and surgical procedures (Al‐Khateeb, Al‐Hadi Hamasha, and Ababneh 2007; Ghandourah et al. 2023).

The MF is an oblique opening on the external surface of the mandible, serving as a passageway for the mental nerve, artery, and vein (Ceballos et al. 2017; Pancer, Garaicoa‐Pazmiño, and Bashutski 2014; Imada et al. 2014). The mental nerve, a branch of the inferior alveolar nerve, plays a crucial role in providing sensation to the lower lip, buccal vestibule, and gingiva (Chkoura and El Wady 2013). The location of the MF can vary widely; typically, it is found between the apices of the roots of the lower first and second premolars or below the root of the second premolar. However, it can range from as far anterior as the canine to as far posterior as the first molar (Sawyer, Kiely, and Pyle 1998; Currie et al. 2016).

The position of the MF can vary greatly among individuals of the same ethnicity, gender, or age. Additionally, in the same person, the position of the MF can differ on each side of the mandible (Mahabob et al. 2021; Robinson and Yoakum 2020). Additionally, the MF can exist as a single opening or show variations, such as double, multiple, or, in rare cases, may be completely absent (Robinson and Yoakum 2020).

Accurate identification of the position of the MF is of utmost importance to prevent injuries to the mental nerve during various procedures. These procedures include the administration of local anesthesia, treatment of fractures in the parasymphyseal area, orthognathic surgery, implant placement, and the placement of complete dentures, especially on severely resorbed edentulous ridges. Additionally, accurate identification is crucial during endodontic, periapical, and periodontal surgeries (Mohammad et al. 2016; Phillips, Weller, and Kulild 1990). Failure to correctly locate the MF can result in complications such as sensory disturbances, formation of traumatic neuromas, bleeding, and bruising (Wadhwani et al. 2008; Yeong‐Hoon and Hun‐Mu 2015; Renton et al. 2012).

Several techniques have been explored to accurately locate the MF, including visual observation during surgery, cadaver dissection, periapical and panoramic X‐rays, computed tomography (CT), cone‐beam computed tomography (CBCT) (Alqutaibi, Alghauli, et al. 2024, Alqutaibi et al. 2022; Borzangy et al. 2023), and magnetic resonance imaging (MRI) (Chau 2012). However, these methods have their drawbacks, such as high costs, surgical trauma, radiation exposure, and potential magnification or distortion (Aminoshariae, Su, and Kulild 2014). Nevertheless, studies have shown that digital panoramic radiographs are reasonably accurate and cost‐effective for this purpose (Peker et al. 2009; Yosue and Brooks 1989a). Panoramic radiographs are convenient and cost‐effective for interpretation (Bou Serhal et al. 2002). Furthermore, digital enhancements have been found to significantly improve the quality of these radiographs (Halwani and Muteq 2018).

Al‐Al‐Khateeb, Al‐Hadi Hamasha, and Ababneh (2007) examined panoramic radiographs of individuals from Jordan to analyze the location, form, and appearance characteristics of the MF. The findings indicated that in this specific group of Jordanians, the MF on panoramic radiographs was typically situated below and between the premolar teeth in the lower jaw, and the most common observation was the continuous form.

Ghandourah et al. (2023) studied the horizontal position of the MF in a group of Saudi individuals using digital panoramic radiographs. They compared gender, age, and bilateral symmetry. The study revealed that the MF's location was more strongly associated with the mandibular second premolar than the first premolar. Moreover, bilateral symmetry was observed in 65% of the participants. No statistically significant differences were found between genders.

Ceballos et al. (2017) conducted a review of the literature to determine how often and where the MF appears in panoramic radiographs among different ethnic groups. The review found that the MF was present in 4824 hemi‐mandibles (95.2%), with a slightly higher occurrence on the left side (50.29%) compared to the right side (49.71%). The most common location for the MF was between the roots of the lower premolars (42.22%), near the root of the second lower premolar (33.98%), or behind the root of the second lower premolar (10.98%).

This study aimed to analyze various characteristics of the MF. These characteristics include its vertical and horizontal positions, shape, appearance, and position in relation to the mandibular canal. Additionally, the presence of accessory mental foramina was examined. The study also assessed whether the appearance of the MF on the panoramic image mimicked or did not mimic a periapical radiolucent lesion when they were at or close to the apices of the teeth' roots. Furthermore, the study assessed the differences between genders and mandibular sides and also evaluated bilateral symmetry.

## 2 Materials and Methods

This study, which was conducted at the Faculty of Dentistry, Ibb University in Yemen, is a descriptive study that utilized a retrospective analysis to investigate the characteristics of the MF using digital panoramic radiographs. The primary objective of this study was to examine MF variations between genders, assess bilateral symmetry, and compare features on different sides of the mandible. Ethical approval for the study was obtained from the College of Dentistry, Taibah University, Al Madinah, Saudi Arabia, and the data collection period spanned from July 2022 to April 2023.

The radiographs used in this study were obtained from the archives of the Department of Oral Radiology at the Faculty of Dentistry. To ensure the reliability of the observations, four dental examiners were involved in both the collection and analysis of the radiographs. Before the main analysis, a calibration exercise was conducted using 20 panoramic radiographs that were selected with the guidance of a consultant in oral and maxillofacial surgery (F.A.). Each examiner conducted the calibration individually, resulting in measurements that showed over 90% reproducibility.

The inclusion criteria specified that only clear panoramic radiographs with good visibility of anatomical landmarks would be considered. These radiographs had to be from individuals aged 18 to 50 years, of both genders, and with all permanent canines to first molars present bilaterally. Exclusion criteria were established to eliminate radiographs that displayed artifacts, lesions in the regions of canines, premolars, and first molars, presence of orthodontic appliances, any history of orthognathic surgery or orthodontic treatment, crowns or bridges, missing teeth, or any history of mandibular fractures involving the parasymphyseal region and dental implants in the anterior or premolar areas.

The radiographic equipment used was a Vatech Pax‐400C X‐Ray Machine from Seogu‐dong, Korea. The exposure parameters were adjusted based on the patient's age and size, ranging from 40 to 90 kVp, 2–10 mA, and an exposure time of 13–15 s. Vatech EzDent‐iTM Software was used for the visualization and assessment of the MF characteristics. Out of the initial pool of 832 panoramic radiographs, only 500 (237 males and 263 females) met the inclusion criteria. The remaining 332 were excluded due to noncompliance with the set criteria. We implemented a comprehensive visual examination of each radiograph to detect common artifacts, including those resulting from patient movement, incorrect positioning, or technical issues during image acquisition.

In cases where multiple foramina were present, the uppermost foramen closest to the mandibular canal was considered as the main foramen, following the approach described by Yosue and Brooks (1989b). A purposive sampling method was used to select these 500 participants for detailed analysis, as outlined in Table 1 of the study.

**Table 1:** Scoring criteria for assessing the positions and other characteristics of the mental foramen.

Antero‐posterior (horizontal) position of the MF was classified into six positions (Tebo and Telford 1950) (Figure 1A)			Superio‐inferior (vertical) relationships between MF and root apices of the lower premolars were classified into three positions (Sekerci et al. 2013) (Figure 1B)	
*Position I*	MF between the canine and the first premolar		*Position I*	**MF positioned above the level of apices of the lower premolars**
*Position II*	MF situated in line with the first premolar		*Position II*	**MF positioned at the level of apices of the lower premolars**
*Position III*	MF between the first and second premolars		*Position III*	**MF positioned below the level of apices of the lower premolars**
*Position IV*	MF situated in line with the second premolar		**The appearance of the MF in relation to the mandibular canal** (Yosue and Brooks 1989b) (Figure 2)	
*Position V*	MF between the second premolar and the first molar		*Continuous*	**The MF is continuous with the mandibular canal**
*Position VI*	MF situated in line with the first molar		*Separated*	**The MF is distinctly separated from the mandibular canal and appears as a well‑defined radiolucency with a distinct border**
**The shape of the MF was assessed** (Popović et al. 2017) (Figure 2B,C)			*Diffuse*	**The MF has an indistinct border**
			*Unidentified*	**The MF is undetectable**
Round	Oval	Irregular	**The position of the MF in relation to the mandibular canal** (Petrovski et al. 2020) (Figure 2)	
**The presence of accessory mental foramina**				
1 foramen	2 foramina		*Ascending*	**The mandibular canal has an ascending path toward the MF**
**MF mimic or did not mimic periapical pathology**			*Descending*	**The mandibular canal descends directly from the area of the second lower molar to the MF**
*Mimic*	MF mimics in appearance the periapical radiolucent lesion		*Horizontal*	**Horizontal orientation of the mandibular canal toward the MF**
*Didn't mimic*	MF did not mimic in appearance the periapical radiolucent lesion		*Wavy*	**A wavy motion of the mandibular canal throughout the mandible**

**Figure 1:**
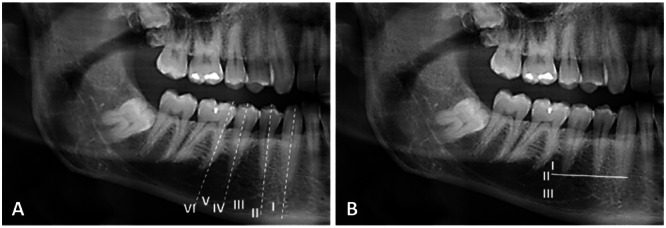
Cropped panoramic images showing the horizontal (A) and vertical (B) positions of the mental foramen.

**Figure 2:**
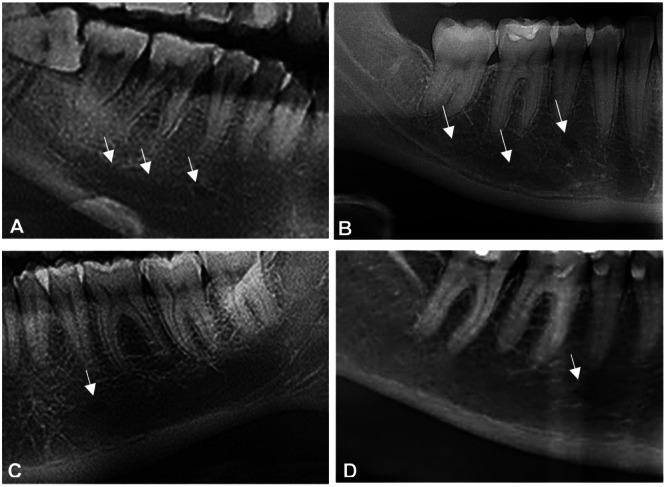
Appearance of the mental foramen on cropped panoramic images. (A) Continuous relation and horizontal path; (B) oval shape, separated relation and descending path; (C) round shape and diffuse; and (D) unidentified type.

In order to evaluate the horizontal position of the MF, vertical lines were established that ran parallel to the long axis of the lower canine, first and second premolars, and the first molar. Subsequently, the horizontal location of the MF was recorded based on these vertical markers, as illustrated in Figure 1A. Additionally, to assess the vertical position of the MF, a horizontal line was drawn at the level of the apex of the lower first and second premolars. The positioning of the foramen with respect to this line was documented, as depicted in Figure 1B. Figure 2 illustrates the shape of the MF and its appearance and position in relation to the mandibular canal.

The analysis also included whether the appearance of the MF resembled or did not resemble a radiolucent pathology and the examination of accessory mental foramina. Data were collected and analyzed using SPSS software. The statistical significance between the variables was assessed using the chi‐square test, with a significance level set at *p* < 0.05.

## 3 Results

The analysis encompassed a comprehensive collection of 500 panoramic images, which accurately represented 1000 sides, fulfilling the predetermined inclusion criteria. The age range of the patients varied from 18 to 50 years, with an average age of 34 years. The sample population comprised of 237 males (47.4%) and 263 females (52.6%). Notably, no statistically significant disparities were identified between genders.

### 3.1 Antero‐Posterior (Horizontal) Position of the MF

The most common horizontal position of the mental foramen (MF) was position III (63.2%), which corresponds to the position of the MF between the first and second lower premolars. This was followed by position IV (28.3%), where the MF is aligned with the long axis of the second lower premolar. Furthermore, position III was more commonly observed in both genders. There was no significant difference between the right and left MF or between males and females regarding horizontal position (*p* > 0.05) (Table 2).

**Table 2:** Comparison of the horizontal position of the mental foramen in relation to gender and sides (*n* = 1000 for both sides).

Horizontal position of the MF	Right side			Left side			Grand total, *n* (%)
	Total, *n* (%)	Male, *n* (%)	Female, *n* (%)	Total, *n* (%)	Male, *n* (%)	Female, *n* (%)	
**Position I**	3 (0.6)	1 (0.4)	2 (0.76)	2 (0.4)	1 (0.4)	1 (0.4)	5 (0.5)
**Position II**	13 (2.6)	9 (3.8)	4 (1.5)	13 (2.6)	5 (2.1)	8 (3)	26 (2.6)
**Position III**	300 (60)	142 (59.9)	158 (60)	332 (66.4)	163 (68.8)	169 (64.3)	632 (63.2)
**Position IV**	157 (31.4)	79 (33.3)	78 (29.6)	126 (25.2)	58 (24.5)	68 (25.9)	283 (28.3)
**Position V**	26 (5.2)	6 (2.5)	20 (7.6)	27 (5.4)	10 (4.2)	17 (6.5)	53 (5.3)
**Position VI**	1 (0.2)	0 (0)	1 (0.4)	0 (0)	0 (0)	0 (0)	1 (0.1)
**Total**	500 (100)	237 (47.4)	263 (52.6)	500 (100)	237 (47.4)	263 (52.6)	1000 (100)
***p*‐value:** Sides	0.72						
***p*‐value:** Gender	Right side: 0.08, Left side: 0.84						

### 3.2 Superio‐Inferior (Vertical) Position of the MF

The most common vertical position of the MF was position III (66.2%), with the MF positioned below the level of the lower premolar apices. This was followed by position II (21.5%), where the MF was positioned at the same level as the lower premolar apices. When considering gender, the most prevalent position was side to side, with both genders showing position III. No differences were observed between sides or between genders in terms of the more frequent vertical position (*p* > 0.05) (Table 3).

**Table 3:** Comparison of the vertical position of the mental foramen in relation to gender and sides (*n* = 1000 for both sides).

Vertical position of the MF	Right side			Left side			Grand total, *n* (%)
	Total, *n* (%)	Male, *n* (%)	Female, *n* (%)	Total, *n* (%)	Male, *n* (%)	Female, *n* (%)	
**Position I**	59 (11.8)	25 (10.5)	34 (12.9)	64 (12.8)	26 (11)	38 (14.4)	123 (12.3)
**Position II**	115 (23)	58 (24.5)	57 (21.7)	100 (20)	51 (21.5)	49 (18.6)	215 (21.5)
**Position III**	326 (65.2)	154 (65)	172 (65.4)	336 (67.2)	160 (67.5)	176 (67)	662 (66.2)
**Total**	500 (100)	237 (47.4)	263 (52.6)	500 (100)	237 (47.4)	263 (52.6)	1000 (100)
***p*‐value:** Sides	0.49						
***p*‐value:** Gender	Right side: 0.6, Left side: 0.43						

### 3.3 The Shape of the MF

The most prevalent shape for the MF was round (46.3%), followed by irregular shape (38.2%). The round shape was more common in both genders and on both sides. No significant differences were observed between the sides in terms of shape (*p* > 0.05). However, a significant difference (*p* < 0.05) was found in terms of gender on the right side, with the round shape being more frequent in females and irregular shape being more frequent in males (Table 4).

**Table 4:** Comparison of the shape of the mental foramen in relation to gender and sides (*n* = 1000 for both sides).

MF shape	Right side			Left side			Grand total, *n* (%)
	Total, *n* (%)	Male, *n* (%)	Female, *n* (%)	Total, *n* (%)	Male, *n* (%)	Female, *n* (%)	
**Round**	224 (44.8)	105 (44.3)	119 (45.2)	239 (47.8)	108 (45.6)	131 (49.8)	463 (46.3%)
**Oval**	77 (15.4)	28 (11.8)	49 (18.6)	78 (15.6)	37 (15.6)	41 (15.6)	155 (15.5%)
**Irregular**	199 (39.8)	104 (43.9)	95 (36.1)	183 (36.6)	92 (38.8)	91 (34.6)	382 (38.2%)
**Total**	500 (100)	237 (47.4)	263 (52.6)	500 (100)	237 (47.4)	263 (52.6)	1000 (100)
***p*‐value:** Sides	0.55						
***p*‐value:** Gender	Right side: 0.05*, Left side: 0.58						

### 3.4 The Appearance of the MF With the Mandibular Canal

The majority of cases (72%) demonstrated a continuous association with the mandibular canal, followed by a separate pattern (19.2%). The consistent association of the MF with the mandibular canal was more prevalent in both males (72.1% on the right side and 69.6% on the left side) and females (73.8% on the right side and 72.2% on the left side). No significant differences were observed between sides or genders regarding the appearance of the MF with the mandibular canal (*p* > 0.05) (Table 5).

**Table 5:** Comparison of appearance of the mental foramen with the mandibular canal in relation to gender and sides (*n* = 1000 for both sides).

Appearance of the MF with the mandibular canal	Right side			Left side			Grand total, *n* (%)
	Total, *n* (%)	Male, *n* (%)	Female, *n* (%)	Total, *n* (%)	Male, *n* (%)	Female, *n* (%)	
Continuous	365 (73)	171 (72.1)	194 (73.8)	355 (71)	165 (69.6)	190 (72.2)	720 (72)
Separated	88 (17.6)	46 (19.4)	42 (16)	104 (20.8)	49 (20.7)	55 (20.9)	192 (19.2)
Diffuse	47 (9.4)	20 (8.4)	27 (10.3)	41 (8.2)	23 (9.7)	18 (6.8)	88 (8.8)
Unidentified	0 (0)	0 (0)	0 (0)	0 (0)	0 (0)	0 (0)	0 (0)
Total	500 (100)	237 (47.4)	263 (52.6)	500 (100)	237 (47.4)	263 (52.6)	1000 (100)
*p*‐value: Sides	0.59						
*p*‐value: Gender	Right side: 0.72, Left side: 0.71						

### 3.5 The Position of the MF With the Mandibular Canal

The predominant orientation (75.6%) of the MF in relation to the mandibular canal was a descending trajectory toward the MF. The descending trajectory was more prevalent in both males (78% on the right side and 74.3% on the left side) and females (76.8% on the right side and 73.4% on the left side). No significant differences were noted between the sides regarding the position of the MF in relation to the mandibular canal (*p* > 0.05). However, a significant difference (*p* < 0.05) was found in terms of gender on the right side, with an ascending pattern being more common in males, whereas other patterns were more frequent in females (Table 6).

**Table 6:** Comparison of the position of the mental foramen with the mandibular canal in relation to gender and sides (*n* = 1000 for both sides).

Position of the MF with the mandibular canal	Right side			Left side			Grand total, *n* (%)
	Total, *n* (%)	Male, *n* (%)	Female, *n* (%)	Total, *n* (%)	Male, *n* (%)	Female, *n* (%)	
Ascending	14 (2.8)	12 (5.1)	2 (0.8)	13 (2.6)	5 (2.1)	8 (3)	27 (2.7)
Descending	387 (77.4)	185 (78)	202 (76.8)	369 (73.8)	176 (74.3)	193 (73.4)	756 (75.6)
Horizontal	46 (9.2)	19 (8)	27 (10.3)	53 (10.6)	27 (11.4)	26 (9.9)	99 (9.9)
Wavy	53 (10.6)	21 (8.9)	32 (12.2)	65 (13)	29 (12.2)	36 (13.7)	118 (11.8)
Total	500 (100)	237 (47.4)	263 (52.6)	500 (100)	237 (47.4)	263 (52.6)	1000 (100)
*p*‐value: Sides	0.81						
*p*‐value: Gender	Right side: 0.02*, Left side: 0.87						

### 3.6 The Presence of Accessory Mental Foramina

A total of 38 accessory mental foramina were identified in 3.8% of the cases examined. Among these, 4.2% were found on the right side and 3.4% on the left side. Statistical analysis showed no significant differences in the frequency of accessory mental foramina between the right and left sides or between genders (Table 7).

**Table 7:** Comparison of the presence of accessory mental foramina in relation to gender and sides (*n* = 1000 for both sides).

Presence of accessory MF	Right side			Left side			Grand total, *n* (%)
	Total, *n* (%)	Male, *n* (%)	Female, *n* (%)	Total, *n* (%)	Male, *n* (%)	Female, *n* (%)	
**1 foramen**	479 (95.8)	226 (95.4)	253 (96.2)	483 (96.6)	228 (96.2)	255 (97)	962 (96.2)
**2 foramina**	21 (4.2)	11 (4.6)	10 (3.8)	17 (3.4)	9 (3.8)	8 (3)	38 (3.8)
**Total**	500 (100)	237 (47.4)	263 (52.6)	500 (100)	237 (47.4)	263 (52.6)	1000 (100)
***p*‐value:** Sides	0.5						
***p*‐value:** Gender	Right side: 0.64, Left side: 0.64						

### 3.7 The Appearance of the MF Was Mimic or did not Mimic a Periapical Radiolucent Pathology

In a total of 77 radiographs, the manifestation of the MF displayed similarities with periapical radiolucent lesions in approximately 7.7% of cases. Statistical analysis revealed no significant differences between sides or between genders in terms of the occurrence of MF that either mimicked or did not mimic periapical pathology (Table 8).

**Table 8:** Comparison of the position and appearance of the mental foreman on whether it mimicked or did not mimic periapical pathology in relation to gender and sides (*n* = 1000 for both sides).

MF mimicked or did not mimic periapical pathology	Right side			Left side			Grand total, *n* (%)
	Total, *n* (%)	Male, *n* (%)	Female, *n* (%)	Total, *n* (%)	Male, *n* (%)	Female, *n* (%)	
**Mimicked**	40 (8)	19 (8)	21 (8)	37 (7.4)	21 (9)	16 (6)	77 (7.7)
**Did not mimic**	460 (92)	218 (92)	242 (92)	463 (92.6)	216 (91)	247 (94)	923 (92.3)
**Total**	500 (100)	237 (47.4)	263 (52.6)	500 (100)	237 (47.4)	263 (52.6)	1000 (100)
***p*‐value:** Sides	0.72						
***p*‐value:** Gender	Right side: 0.98, Left side: 0.23						

### 3.8 Bilateral Symmetry of the MF

**Figure 3:**
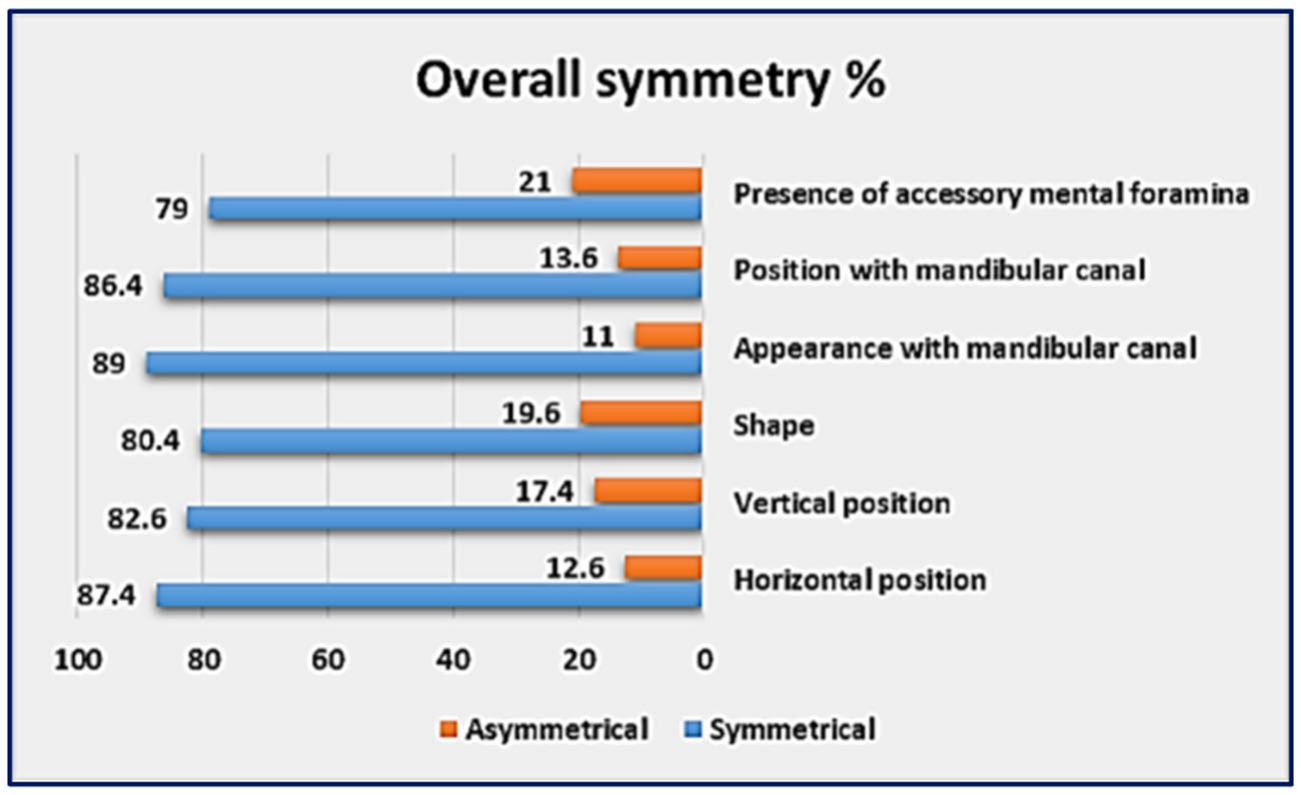
Bar chart showing the overall symmetry of the radiographic characteristics of the MF for both sides of the mandible.

In terms of the symmetry of the MF, the horizontal position was found to be symmetrical in 87.4% of cases and asymmetrical in 12.6% of cases. The vertical position showed bilateral symmetry in 82.6% of cases, whereas asymmetry was observed in 17.4% of cases. The shape of the MF was symmetrical in 80.4% of cases and asymmetrical in 19.6% of cases. The configuration of the MF in relation to the mandibular canal was symmetrical in 89% of cases and asymmetrical in 11% of cases. Additionally, the position of the MF with respect to the mandibular canal was symmetrical in 86.4% of cases and asymmetrical in 13.6% of cases. The presence of accessory mental foramina was found to be symmetrical in 79% of cases and asymmetrical in 21% of cases. The symmetry of each feature is summarized in Table 9, and the overall symmetry is depicted in Figure 3.

**Table 9:** Distribution of symmetrical and asymmetrical features of MF.

Horizontal position of the MF							
The symmetry *n* (%)	Class I	Class II	Class III	Class IV	Class V	Class VI	Total
Symmetrical	2 (0.4)	5 (1)	296 (59.2)	122 (24.4)	12 (2.4)	0 (0)	437 (87.4)
Asymmetrical	1 (0.2)	2 (0.4)	38 (7.6)	18 (3.6)	122 (24.4)	0 (0)	63 (12.6)
**Vertical position of the MF**							
The symmetry, *n* (%)	**Class I**	**Class II**	**Class III**	**Total**			
Symmetrical: *n* (%)	44 (8.8)	92 (18.4)	277 (55.4)	413 (82.6)			
Asymmetrical: *n* (%)	10 (2)	11 (2.2)	66 (13.2)	87 (17.4)			
**MF shape**							
The symmetry, *n* (%)	**Round**	**Oval**	**Irregular**	**Total**			
Symmetrical: *n* (%)	181 (36.2)	60 (12)	161 (32.2)	402 (80.4)			
Asymmetrical: *n* (%)	43 (8.6)	17 (3.4)	38 (7.6)	98 (19.6)			
**Appearance of the MF with the mandibular canal**							
The symmetry *n* (%)	**Continuous**	**Separated**	**Diffuse**	**Unidentified**	**Total**		
Symmetrical: *n* (%)	331 (66.2)	76 (15.2)	38 (7.6)	0 (0)	445 (89)		
Asymmetrical: *n* (%)	29 (5.8)	20 (4)	6 (1.2)	0 (0)	55 (11)		
**Position of the MF with the mandibular canal**							
The symmetry, *n* (%)	**Ascending**	**Descending**	**Horizontal**	**Wavy**	**Total**		
Symmetrical: *n* (%)	13 (2.6)	349 (69.8)	29 (5.8)	41 (8.2)	432 (86.4)		
Asymmetrical: *n* (%)	2 (0.4)	30 (6)	19 (3.8)	17 (3.4)	68 (13.6)		
**Presence of accessory MF**							
The symmetry, *n* (%)	**2 foramina**						
Symmetrical: *n* (%)	15 (79)						
Asymmetrical: *n* (%)	8 (21)						

## 4 Discussion

Panoramic radiography is extensively used in maxillofacial imaging for diagnostic, screening, and procedural planning purposes (Ari et al. 2005). It serves as a reliable method to determine the position of the MF, which is essential for both diagnostic and surgical interventions involving the mandible. However, caution must be exercised when relying on panoramic radiographs for precise measurements and comparisons (Suragimath, Suragimath, and Murlasiddiah 2016). Research conducted with dry skulls has shown a strong correlation between the radiographic and actual positions of the MF (Phillips, Weller, and Kulild 1990; Wang et al. 1986).

Despite advances in diagnostic technologies, including artificial intelligence and 3D imaging (Al‐Sarem et al. 2022; Alqutaibi, Algabri, et al. 2024, Alqutaibi et al. 2023), panoramic X‐rays remain the mainstay for dental evaluations in many low‐income countries. Despite their limitations, such as image distortion and anatomical overlap, panoramic X‐rays continue to provide reliable results for a wide range of dental issues. This is especially important in economically constrained regions with limited access to state‐of‐the‐art technologies. Panoramic X‐rays offer a cost‐effective, accessible, and straightforward approach to obtaining comprehensive views of the dental arches, mandible, and maxilla, enabling initial assessment and ongoing management of dental conditions. However, dental professionals in these areas need specialized skills to interpret panoramic images despite their limitations. This highlights the global disparities in healthcare technology access and underscores the vital role of basic imaging modalities in promoting oral health in resource‐limited settings.

In our study, panoramic radiographs were selected primarily because they provide a crucial diagnostic view for general dentists and specialists, particularly in minor oral surgeries and other dental procedures. These radiographs offer a comprehensive overview of the teeth, their supporting structures, and the surrounding anatomy in a single image. This facilitates the evaluation of tooth placement, detection of pathology, and identification of anatomical landmarks such as the MF. Therefore, our research plays a vital role in enhancing the understanding of MF anatomy, enabling dental professionals to perform various procedures with increased precision and safety. Despite the detailed three‐dimensional data provided by CBCT, the higher radiation exposure and cost associated with its use often make panoramic radiographs a more practical choice for routine assessments and initial treatment planning.

Neves et al. (2014) compared panoramic radiography to CBCT in identifying anatomical variations in the mandibular canal and MF. They analyzed 127 preoperative images from each modality, reviewed by two oral and maxillofacial radiologists. CBCT outperformed panoramic radiographs in detecting additional mental foramina and bifid mandibular canals (7.4% and 9.8% vs. 1.2% and 7.4%, respectively). However, the disparities in mandibular canal variations identified by the two modalities were not statistically significant (*p* > 0.05). The study concluded that CBCT provides better visualization, but panoramic radiography remains a viable option for identifying bifid mandibular canals, especially when CBCT is unavailable.

Our analysis of 500 digital panoramic radiographs demonstrated the bilateral presence of the MF in all cases, consistent with previous studies conducted on both dry skulls (Singh and Srivastav 2010; Oliveira Junior et al. 2009; Voljevica 2015) and panoramic radiographs (Al‐Khateeb, Al‐Hadi Hamasha, and Ababneh 2007; Ghandourah et al. 2023; Suragimath, Suragimath, and Murlasiddiah 2016). The existing literature suggests that the absence of the MF is extremely rare (Greenstein and Tarnow 2006; Ulu et al. 2016). The study also revealed variations in the horizontal placement of the MF, with Position III, predominantly located between the premolars, and Position IV, aligned with the second premolar, being the most frequent positions. These findings are in agreement with prior research (Al‐Khateeb, Al‐Hadi Hamasha, and Ababneh 2007; Chkoura and El Wady 2013; Halwani and Muteq 2018; Zmyslowska‐Polakowska et al. 2019; Abed et al. 2016; Bosykh et al. 2019; Al‐Mahalawy et al. 2017; Alrahabi and Zafar 2018; Han, Hwang, and Jeong 2016; Muinelo‐Lorenzo et al. 2015). Notably, it was observed that position III was more prevalent among females.

Factors that influence the position of the MF include mandibular growth, differential rates of bone and periosteum development, and the morphological characteristics of the foramen itself (Philips, Weller, and Kulild 1992). However, it is important to note that inherent distortions and magnification in panoramic radiography can result in some unavoidable variation in perceived position (Ramstad et al. 1978).

In terms of vertical position, our study found that the most common position of the MF (66.2%) was below the apices of the lower premolars' roots, regardless of gender or age. The second most frequent position was at the level of these roots (21.5%). These findings are consistent with previous studies that utilized CBCT (Sekerci et al. 2013) and panoramic radiography (Al‐Khateeb, Al‐Hadi Hamasha, and Ababneh 2007) and other researchers who utilized both CBCT and panoramic radiograph methods (Alam et al. 2018; Khalifa 2022). Variations in vertical position are influenced by factors such as root length and age, and significant changes in position can occur from childhood to adulthood, particularly following tooth eruption or loss (Solomon 2011).

With advancing age, there is an increased frequency of the MF being located more posteriorly and inferiorly (Al‐Khateeb, Al‐Hadi Hamasha, and Ababneh 2007; Santini and Land 1990). This shift may be related to anterior tooth drift due to age‐related attrition (Al‐Khateeb, Al‐Hadi Hamasha, and Ababneh 2007). The position of the MF varies in the superior–inferior dimension; although the length of roots contributes to this variability, age is a significant factor. In children, the foramen is closer to the alveolar margin before tooth eruption, and then shifts to a midpoint between the alveolar and inferior margins during eruption. In adults with the teeth preserved, it is closer to the inferior border, but with tooth loss and bone resorption, the alveolar crest moves downward, bringing the MF closer to the alveolar margin (Al‐Khateeb, Al‐Hadi Hamasha, and Ababneh 2007).

Our observations also confirmed that the majority of the mental foramina were round, which is consistent with previous studies (Al‐Khateeb, Al‐Hadi Hamasha, and Ababneh 2007; Mohammad et al. 2016; Sekerci et al. 2013; Rai, Shrestha, and Jha 2014), although certain populations have reported a higher prevalence of oval‐shaped foramina (Gershenson, Nathan, and Luchansky 1986; Chu et al. 2014; Igbigbi and Lebona 2005; Parmar et al. 2013). Furthermore, the continuous type was the most frequently observed relationship between the MF and the mandibular canal, which aligns with findings in the existing literature (Mohammad et al. 2016; Yosue and Brooks 1989a, 1989b; Sekerci et al. 2013). Our research findings show that most cases demonstrated a descending path of the mandibular canal, passing through the mandible's body from the second lower molar to the MF. These results are in line with the majority of the existing literature (Petrovski et al. 2020; Jeon et al. 2017; Velasco‐Torres et al. 2017; Khorshidi et al. 2017).

Our study also identified the presence of accessory mental foramina in 3.8% of cases, which is in line with previous literature (Al‐Khateeb, Al‐Hadi Hamasha, and Ababneh 2007; Al‐Qaisi et al. 2023; Shokri, Maleki, and Tapak 2024), but differs significantly from other population studies that reported a higher prevalence of accessory mental foramina (Sekerci et al. 2013; Agthong, Huanmanop, and Chentanez 2005; Berge and Bergman 2001; Capote, Gonçalves, and Campos 2015). This highlights potential clinical implications for surgeries and procedures involving the mental region.

We observed significant symmetry in both the horizontal (87.4%) and vertical (82.6%) positions of the MF, with rates comparable to those reported in various populations (Al‐Khateeb, Al‐Hadi Hamasha, and Ababneh 2007; Ghandourah et al. 2023; Gungor et al. 2006; Ngeow and Yuzawati 2003; Haghanifar and Rokouei 2009). However, these findings also suggest that absolute symmetry in MF positioning is uncommon, highlighting the inherent individual variability. Considering these variabilities, as well as potential distortions caused by magnification and patient positioning, panoramic radiographs should be carefully interpreted, particularly when evaluating mandibular symmetry (Laster et al. 2005; Schulze, Schalldach, and d'Hoedt 2000).

In clinical practice, distinguishing between the MF and periapical pathology can pose challenges, particularly when the foramen aligns with the apices of premolars on radiographs. Confirmation of the true nature of radiolucency often necessitates additional evidence, such as the presence of the mandibular canal or visible lamina dura around the root apex, even in the presence of potential issues related to image clarity due to low density or “burnout” (Huumonen and Ørstavik 2002; Lakshman, Kannepady, and Kalkur 2014). As recommended by the European Society of Endodontology, comprehensive preoperative radiographs should encompass the entire root and any surrounding anatomical structures to ensure accurate diagnosis and treatment planning (European Society of Endodontology 2006; Chong et al. 2017).

One limitation of this study is its reliance on a retrospective analysis of archived digital panoramic radiographs, which may introduce selection bias due to the exclusion criteria and the non‐randomized sampling method. The purposive sampling approach, while ensuring a specific population focus, limits the generalizability of the findings to broader populations. Additionally, dependence on specific radiographic equipment (Vatech Pax‐400C X‐Ray Machine) may have influenced the generalizability. The study also excluded patients with orthodontic appliances, dental restorations, or mandibular fractures, which may overlook the potential influence of these factors on the characteristics of the MF. Lastly, the study's geographic focus on a specific population in Yemen may limit its applicability to populations with different ethnic, genetic, or environmental backgrounds.

## 5 Conclusions

Based on the results of this study, the following conclusions can be drawn:

In this Yemeni study, the position of the MF on panoramic radiographs was commonly below and between the lower premolar teeth horizontally and below the level of the lower premolar apices vertically. Additionally, the majority of cases of the MF showed a round shape and continuous appearance with a descending path relative to the mandibular canal with minimal differences between genders and sides. High levels of symmetry in these characteristics underline the predictable nature of the MF in dental practice, aiding in diagnostic, surgical, and procedural planning. However, variations such as the presence of accessory mental foramina and the occasional appearance of the MF mimicking pathological conditions necessitate careful examination to ensure accurate diagnosis and treatment planning.

## Author Contributions

**Radhwan Algabri and Faisal Abulohoom:** conceptualization, methodology, investigation, results, writing–original draft, writing–review and editing, and project administration and supervision. **Abdelrahman Fadag:** methodology, results, writing–original draft, writing–review, and supervision. **Hesham Al‐Sharani, Sami Al‐Aqab, Nassr Al‐Hutbany, and Emad Alryashi:** methodology, investigation, and results. **Ahmed Keshk:** results, writing–original draft, and writing–review and editing. **Ahmed Yaseen Alqutaibi:** methodology, results, writing–original draft, writing–review, and Supervision.

## Ethics Statement

The Faculty of Dentistry's ethics committee at Taibah University approved the research protocol, identified as reference number #111122.

## Conflicts of Interest

The authors declare no conflicts of interest.

## Clinical Implications

The study's findings on the MF have important direct implications for dentistry and maxillofacial surgery. Healthcare professionals can enhance procedural accuracy, reduce complications, and optimize patient care by utilizing this knowledge in routine and complex dental treatments.

## Data Availability

The data that support the findings of this study are available from the corresponding author upon reasonable request.
